# The atopic dermatitis blood signature is characterized by increases in inflammatory and cardiovascular risk proteins

**DOI:** 10.1038/s41598-017-09207-z

**Published:** 2017-08-18

**Authors:** Patrick M. Brunner, Mayte Suárez-Fariñas, Helen He, Kunal Malik, Huei-Chi Wen, Juana Gonzalez, Tom Chih-Chieh Chan, Yeriel Estrada, Xiuzhong Zheng, Saakshi Khattri, Annunziata Dattola, James G. Krueger, Emma Guttman-Yassky

**Affiliations:** 1 The Laboratory for Investigative Dermatology, The Rockefeller University, New York, NY USA; 2 Department of Dermatology and the Laboratory for Inflammatory Skin Diseases, Icahn School of Medicine at Mount Sinai, New York, NY USA; 3 Department of Population Health Science and Policy, Icahn School of Medicine at Mount Sinai, New York, NY USA; 4 Department of Genetics and Genomics Science, Icahn Institute for Genomics and Multiscale Biology, New York, NY USA

## Abstract

Beyond classic “allergic”/atopic comorbidities, atopic dermatitis (AD) emerges as systemic disease with increased cardiovascular risk. To better define serum inflammatory and cardiovascular risk proteins, we used an OLINK high-throughput proteomic assay to analyze moderate-to-severe AD (n = 59) compared to psoriasis (n = 22) and healthy controls (n = 18). Compared to controls, 10 proteins were increased in serum of both diseases, including Th1 (IFN-γ, CXCL9, TNF-β) and Th17 (CCL20) markers. 48 proteins each were uniquely upregulated in AD and psoriasis. Consistent with skin expression, AD serum showed up-regulation of Th2 (IL-13, CCL17, eotaxin-1/CCL11, CCL13, CCL4, IL-10), Th1 (CXCL10, CXCL11) and Th1/Th17/Th22 (IL-12/IL-23p40) responses. Surprisingly, some markers of atherosclerosis (fractalkine/CX3CL1, CCL8, M-CSF, HGF), T-cell development/activation (CD40L, IL-7, CCL25, IL-2RB, IL-15RA, CD6) and angiogenesis (VEGF-A) were significantly increased only in AD. Multiple inflammatory pathways showed stronger enrichment in AD than psoriasis. Several atherosclerosis mediators in serum (e.g. E-selectin, PI3/elafin, CCL7, IL-16) correlated with SCORAD, but not BMI. Also, AD inflammatory mediators (e.g. MMP12, IL-12/IL-23p40, CXCL9, CCL22, PI3/Elafin) correlated between blood and lesional as well as non-lesional skin. Overall, the AD blood signature was largely different compared to psoriasis, with dysregulation of inflammatory and cardiovascular risk markers, strongly supporting its systemic nature beyond atopic/allergic association.

## Introduction

Atopic dermatitis (AD) is the most common chronic inflammatory skin disease, affecting more than 30 million people in the US^1^. It often begins during early childhood, and adult patients frequently have chronic disease for decades^2–4^. AD has a complex immune milieu with Th2 skewing, but also shows Th22, Th17, and Th1 activation^5,6^. In approximately one third of patients, AD is associated with allergic manifestations (e.g. asthma, food allergies, seasonal allergies)^4,7^. Recently, associations have also been reported with other inflammatory diseases, including rheumatoid arthritis, inflammatory bowel disease^8^, and systemic lupus erythematosus^9^. Emerging data suggests that similar to chronic plaque-type psoriasis, another chronic inflammatory skin disease, AD patients also harbor increased cardiovascular risk factors, including higher rates of cardiovascular disease in some populations^10–15^ that is independent of genetic risk^14^. Consistently, an increased prevalence of coronary artery calcifications is reported in severe AD patients compared to healthy controls^16^.

However, reports on individual comorbid conditions such as stroke or myocardial infarction are controversial and inconsistent^11–14,17^, possibly dependent on contributing factors such as obesity^15,18,19^.

It is currently established that chronic inflammation accelerates atherosclerosis due to repetitive vascular injury^20^. For example, elevated serum levels of TNF-α and IL-17 are thought to contribute to increased cardiovascular risk in chronic plaque psoriasis^21,22^, possibly mediating endothelial damage. *In vitro* data suggests that IL-17 can indeed contribute to pro-inflammatory changes in endothelial cells, and inhibition of IL-17 in a mouse model of atherosclerosis showed significantly ameliorated disease^23,24^. Importantly, many of these “psoriasis” mediators are also found in AD^5,25,26^. Like AD, allergic asthma is a Th2-driven disease with contributions of additional cytokine pathways^27^. Patients with asthma are demonstrated to be at increased risk for atherosclerosis^28^, implying that chronic inflammation across diseases in general, rather than a specific cytokine, may be primarily responsible for increased cardiovascular risk^29^.

Increasing evidence exists for systemic immune activation in AD. Several flow cytometry studies showed increases in activated T-cell subsets in blood from moderate-to-severe AD patients as compared to controls, or psoriasis patients of comparable disease severity^25,30^. Furthermore, several serum biomarkers have been recently reported to correlate with baseline AD severity and/or therapeutic responses. These include Th2 measures such as IL-13, IL-31, CCL17, CCL22, CCL13, Th22-related products (IL-22), Th1-related markers (CXCL10, IFN-γ), E-selectin, IL-16, IL-18, IgE, and eosinophil cationic protein (ECP)^31–38^. Moreover, non-lesional biomarkers show even higher correlations with disease severity compared to lesional biomarkers^33^, also suggesting the systemic nature of AD rather than a disease with only lesional skin-focused inflammation^39^. To characterize a potential systemic inflammatory and cardiovascular risk signature in AD, we assessed respective serum markers using a high throughput proteomic platform. We screened for a large panel of established and exploratory inflammation and cardiovascular risk biomarkers^40^ in serum of moderate-to-severe AD patients with an OLINK Proseek® Multiplex assay that uses proximity extension assay (PEA) technology^41,42^, in comparison to matched controls and moderate-to-severe psoriasis patients. We chose psoriasis as a comparator as it serves as a positive control for systemic inflammation^21^. We show that moderate-to-severe AD patients have increases in multiple inflammatory and cardiovascular risk proteins in serum, that are largely different from increases in blood from psoriasis patients.

## Results

### The proteomic blood signature of AD is largely different from psoriasis

**Figure 1:**
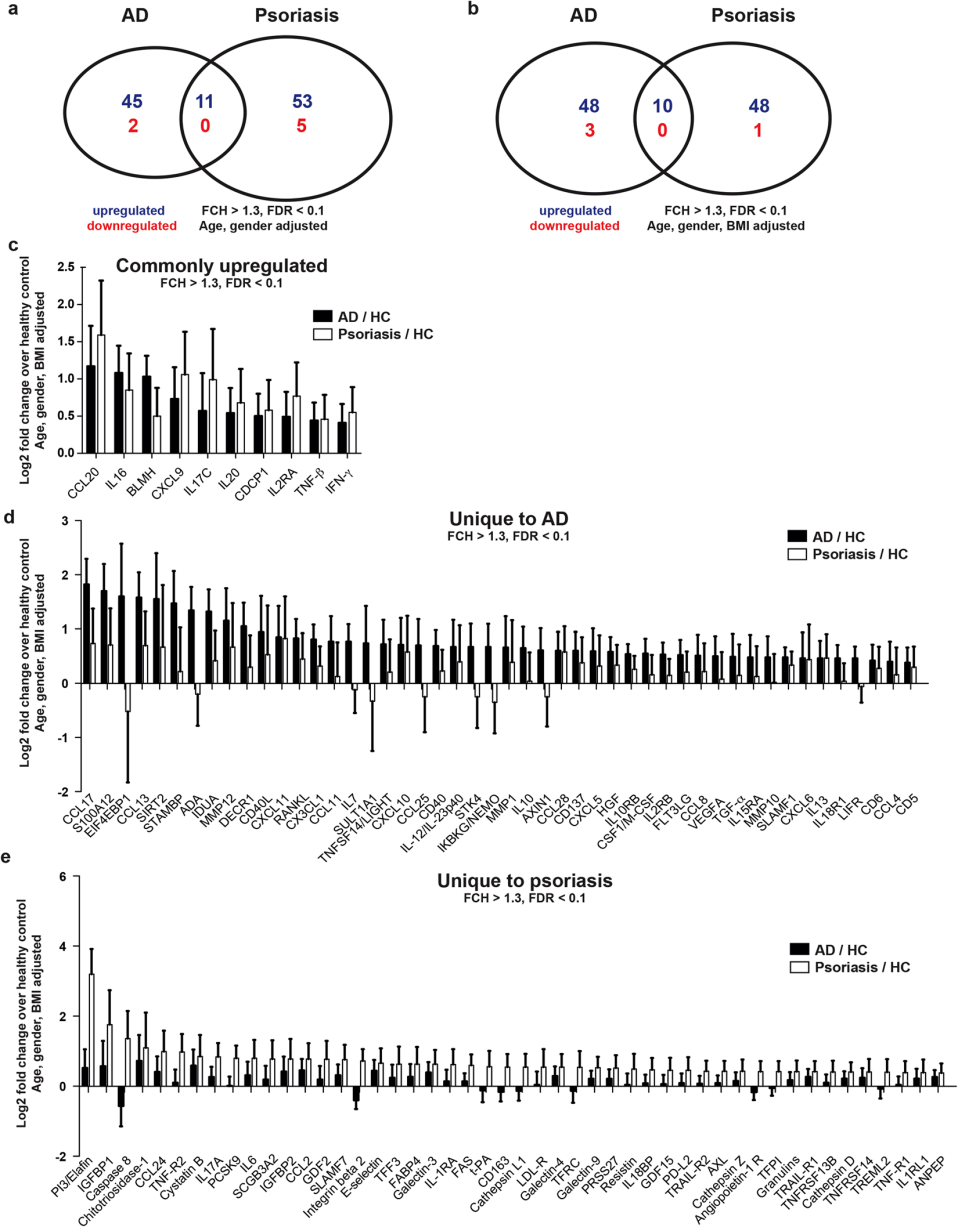
Regulation of inflammatory and cardiovascular risk proteins in AD and psoriasis vs. healthy controls. Venn diagrams of regulated serum proteins compared to healthy controls, adjusted for age/gender **(a)** and age/gender/BMI **(b)**. Markers that were significantly upregulated (FCH > 1.3, FDR < 0.1) in both AD and psoriasis **(c)**, only in AD **(d)** or only in psoriasis **(e)** are depicted as log2 fold change over healthy control serum with their 95% confidence intervals.

Using the OLINK high-throughput proteomic platform, we assessed a panel of 257 immunological and cardiovascular risk proteins in serum of moderate-to-severe AD and psoriasis patients in comparison to healthy control subjects (Table 1). The proximity extension technology used in our study is potentially superior to conventional multiplex immunoassays, since only correctly matched antibody pairs give a signal, yielding higher specificity and sensitivity^40,41^. The body-mass-index (BMI) was similar in AD and controls, while higher in psoriasis patients. Among the 257 investigated proteins, only 11 were significantly upregulated in both diseases when compared to controls (Fig. 1a). The majority of markers were exclusively upregulated in either AD (n = 45) or psoriasis (n = 53), and only a minority were downregulated (Fig. 1a, Supplementary Table S1). When results were adjusted for BMI (Fig. 1b, Supplementary Table S2) and for the presence of asthma and the cardiovascular risk factors such as arterial hypertension, diabetes mellitus, and hypercholesterolemia (Supplementary Figure S1, Supplementary Table S3), the observed differences between AD and psoriasis, compared to controls, were largely preserved. Mutually upregulated proteins included markers of Th1 (IFN-γ, CXCL9, TNF-β/lymphotoxin) and Th17 (CCL20, IL-17C) immune responses, the CD4 T-cell chemoattractant IL-16, and the differentiation/proliferation factor IL-20 (Fig. 1c).

**Table 1:** Summary of demographics and disease severity of study subjects.

	AD	Healthy	Psoriasis	p-value
**Sample size**	n = 59	n = 18	n = 22	
**Age (y) (mean, SD)**	40.5 (15.2)	41.3 (10.3)	46.8 (11.4)	0.208
**BMI (mean, SD)**	27.7 (6.1)	27.6 (4.4)	31.5 (5.2)	*0.031
**SCORAD (mean, SD)**	54.1 (13.2)	NA	NA	
**PASI (mean, SD)**	NA	NA	27.6 (9.9)	
**Gender**				*0.042
Female (n, %)	28 (47.5%)	6 (33.3%)	4 (18.2%)	
Male (n, %)	31 (52.5%)	12 (66.7%)	18 (81.8%)	
**Ethnicity**				*** < 0.001
Asian (n, %)	15 (25.4%)	3 (16.7%)	2 (9.1%)	
African American (n, %)	24 (40.7%)	9 (50.0%)	0 (0.0%)	
Caucasian (n, %)	20 (33.9%)	6 (33.3%)	16 (72.7%)	
**Serum IgE (kU/L) (median, IQR)**	2,412 (5,028)	NA	NA	NA
**Eosinophils (%) (mean, SD)**	6.91 (4.94)	NA	NA	NA
**Asthma bronchiale**				** < 0.001
No	39 (66.1%)	18 (100.0%)	21 (95.5%)	
Yes	20 (33.9%)	0 (0.0%)	1 (4.5%)	
**Arterial hypertension (AT)**				0.759
No	51 (86.4%)	17 (94.4%)	19 (86.4%)	
Yes	8 (13.6%)	1 (5.6%)	3 (13.6%)	
**Diabetes mellitus (DM)**				0.475
No	55 (93.2%)	18 (100.0%)	22 (100%)	
Yes	4 (6.8%)	0 (0.0%)	0 (0.0%)	
**Hypercholesterolemia (HCh)**				0.329
No	52 (88.1%)	18 (100.0%)	19 (86.4%)	
Yes	7 (11.9%)	0 (0.0%)	3 (13.6%)	
**Cardiovascular disease risk factor present (AT and/or DM and/or HCh)**				0.439
No	48 (81.4%)	17 (94.4%)	18 (81.8%)	
Yes	11 (18.6%)	1 (5.6%)	4 (18.2%)	

One-way ANOVA was used for comparisons of means, and Fisher’s exact test was used for comparisons of proportions; *p < 0.05, **p < 0.01, ***p < 0.001; *y years, SD standard deviation, IQR interquartile range*.

AD, but not psoriasis, was characterized by significant upregulation of several additional Th1 (CXCL10, CXCL11), Th2 (IL-13, CCL13, CCL17, CCL11, IL-10), Th17/Th22 (S100A12) and Th1/Th17/Th22 (IL-12/IL-23p40) associated products, as well as a broad array of proteins involved in the development and activation of T-cells (CD40L, IL-7, CCL25, IL2RB, IL15RA, CD6, RANKL, TNFRSF9/CD137) and dendritic cells/DC (CD40, FLT3 ligand) (Fig. 1d). Mediators involved in atherosclerosis (CX3CL1/fractalkine, CCL8, M-CSF, CXCL5, CCL4, HGF), tissue remodeling (MMP-12, MMP-1, MMP-10) and angiogenesis (VEGF-A) were also increased in AD. The growth factor TGF-α, the NF-κB-activator IKBKG/NEMO, molecules mediating chemotaxis of T-cells/B-cells/eosinophils (CCL28) and neutrophils (CXCL5, CXCL6), as well as the soluble cytokine receptors IL-10RB and IL-18R1 were also higher in AD (Fig. 1d).

Proteins significantly upregulated only in psoriasis included “classic” psoriasis markers such as Th17-associated products PI3/Elafin and IL-17A^43^, as well as the angiopoietin receptor (Tie2)^44,45^ (Fig. 1e). Significant increases were noted for molecules involved in coagulation and angiogenesis (t-PA), endothelial activation (IL-6, E-selectin, CCL2), lipid metabolism (FABP4, PCSK9, LDL-R), as well as the adipokine resistin (Fig. 1e). Leptin, which reflects total body adipose tissue^46^, was increased in the BMI-uncorrected cohort (Supplementary Table S1), but was no longer increased in psoriasis after correction for BMI (Fig. 1e, Supplementary Table S2). The IL-1 inhibitor IL-1RA, the macrophage marker CD163 (a hemoglobin-haptoglobin scavenger), several cytokines and soluble cytokine receptors (CCL2, CCL24, TRAIL, TRAIL-R1, TNF-R1, TNF-R2), the IL-18 inhibitor IL-18BP, the angiogenesis inhibitor GDF-2, cardiovascular disease risk proteins (galectin-3, cathepsin L1, cathepsin D, IGFBP2), as well as mediators of cell adhesion (integrin β2, SLAMF7), apoptosis (FAS, Caspase-8), T cell activation (TREML2), proliferation (AXL), co-stimulation (PD-L2), and humoral immunity (TNFRSF13B) were also increased only in psoriasis. In sum, the majority of significantly regulated proteins were different between AD and psoriasis.

### Correlation with skin disease suggests skin-blood-interaction

**Figure 2:**
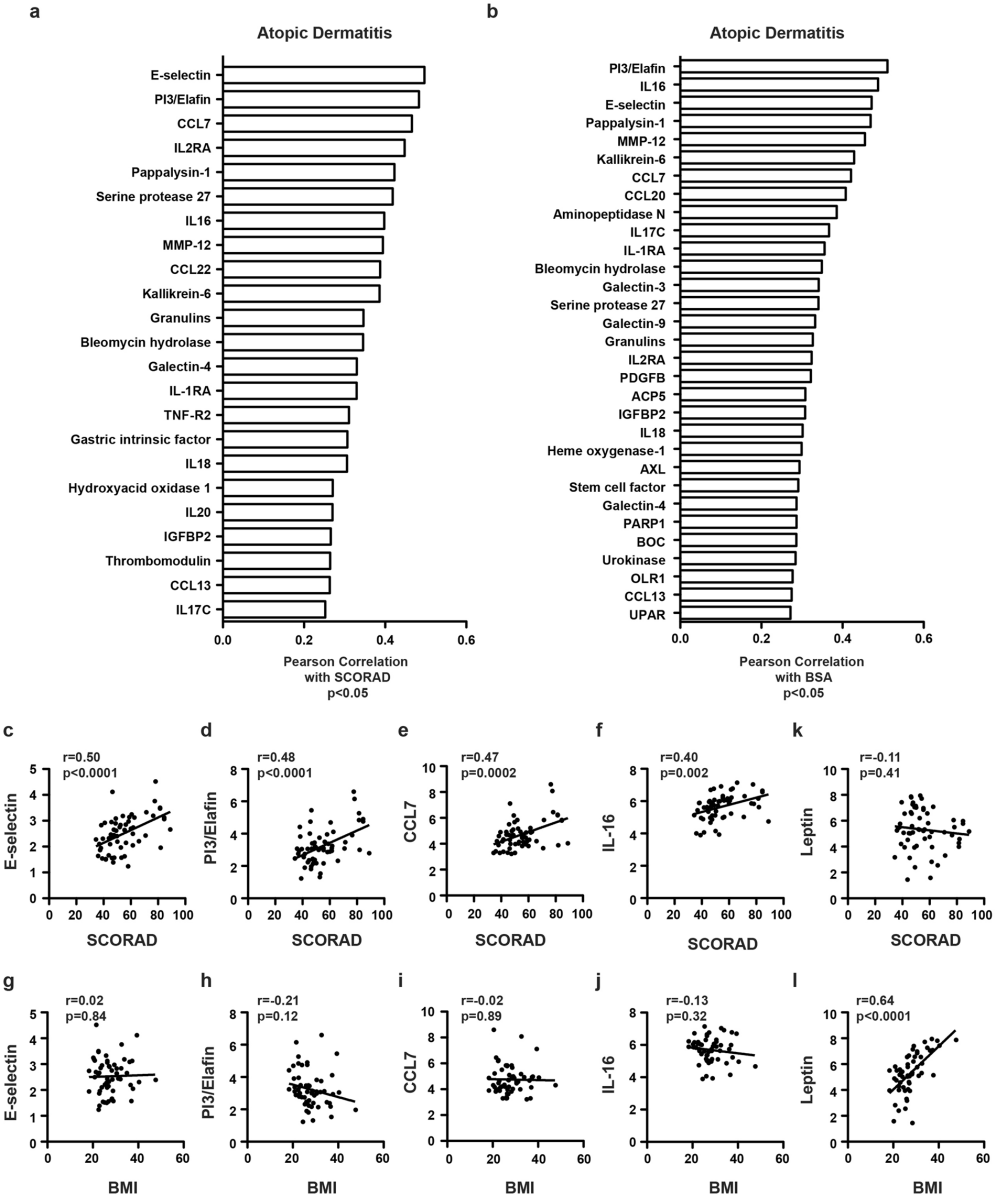
Blood protein correlations with skin disease severity (SCORAD, BSA) and BMI. Pearson correlation coefficients of AD serum proteins significantly correlated with SCORAD **(a)** and body surface area/BSA **(b**), and their respective scatter plots **(c**–**j**), as well as leptin **(k**–**l**), in comparison to correlations with body mass index/BMI.

To investigate whether the extent of AD skin disease influences levels of blood markers, we correlated serum proteins with the clinical AD measures SCORAD (SCORing Atopic Dermatitis, Fig. 2a) and body surface area (BSA) involvement (Fig. 2b). The endothelial cell activation marker E-selectin, the Th17 marker PI3/Elafin, the CCR1/CCR2/CCR3 chemokine CCL7, and the CD4^+^ cell chemoattractant IL-16 showed the most significant correlations for both SCORAD and BSA (Fig. 2a,b). Those serum markers were only correlated with skin scores, but not with body-mass index (BMI) (Fig. 2c–j). Leptin, a measure of total body fat, only correlated with BMI, but not with SCORAD or BSA (Fig. 2k–l), as expected. A complete list of correlation coefficients can be found in Supplementary Table S4.

### Mutual regulation of blood and skin markers

**Figure 3:**
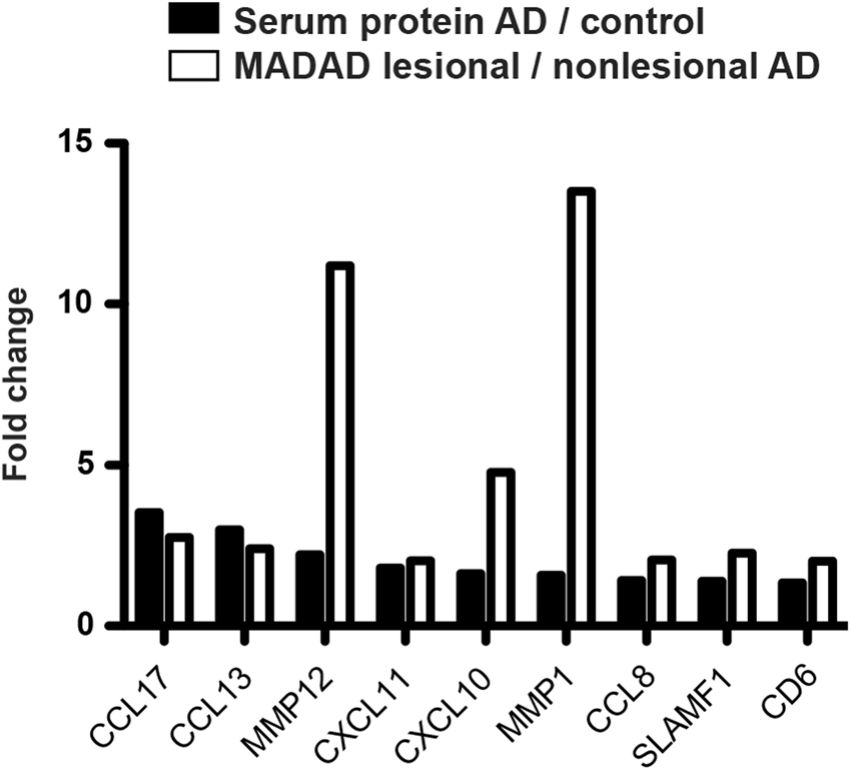
Blood-skin comparisons. Fold change comparisons of AD serum protein levels (AD vs. healthy controls) and skin MADAD transcriptome levels (lesional vs. non-lesional AD).

We next compared blood protein levels in AD with a robust meta-analysis derived atopic dermatitis (MADAD) skin transcriptome that integrates several published cohorts^47^. Markers that were upregulated in both the MADAD transcriptome and the blood were mostly chemokines, as depicted in Fig. 3.

### Correlations of blood inflammatory mediators were found with both lesional and non-lesional AD skin

**Figure 4:**
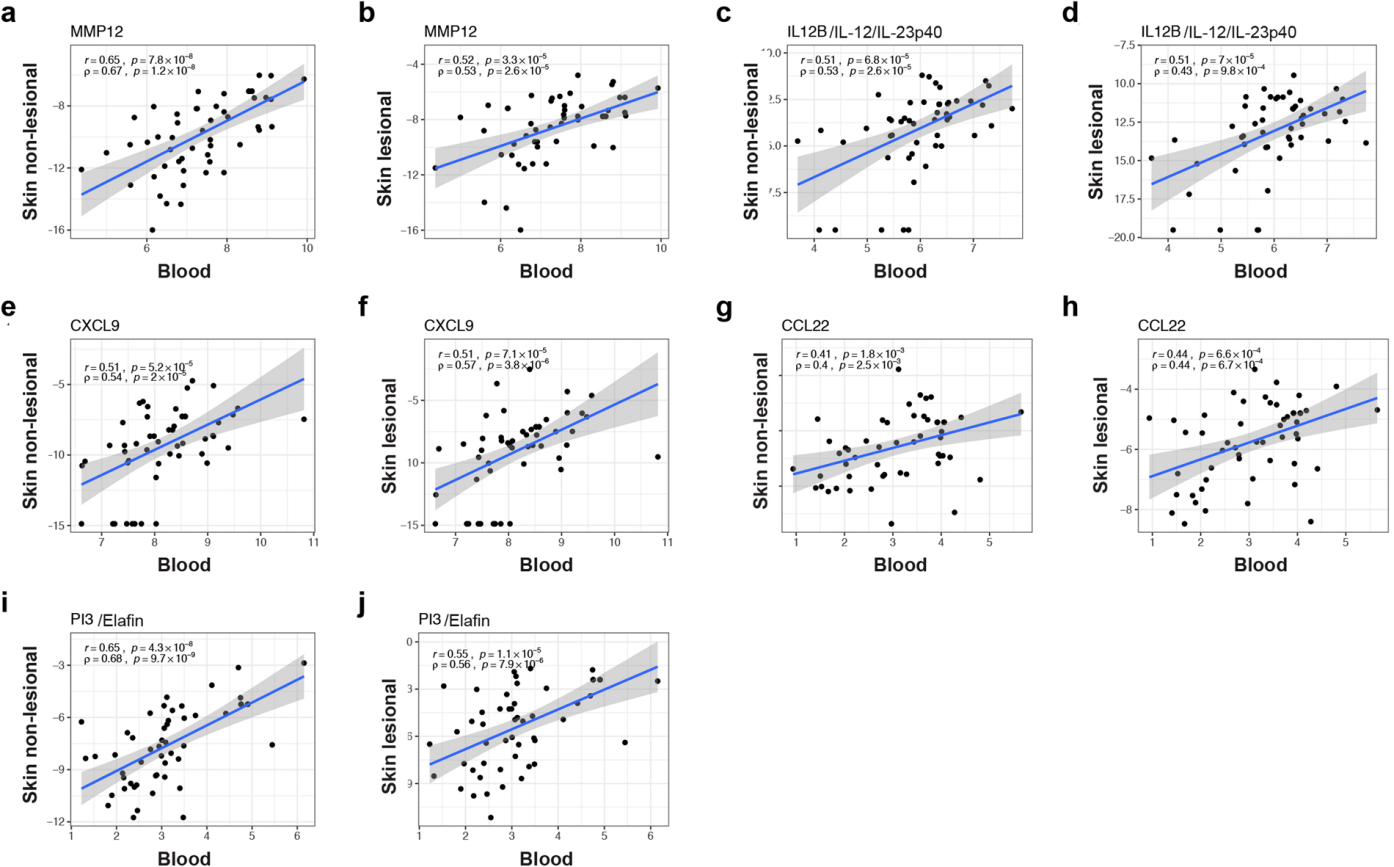
Blood-skin correlations of inflammatory mediators. Correlation plots of selected serum protein levels with their corresponding lesional and non-lesional skin mRNA levels **(a**–**j)**; scatterplots with estimated linear regression and 95% confidence interval; *r Pearson correlation; ρ Spearman correlation.*

As mostly chemokines showed concomitant regulation between lesional skin and serum of AD patients (Fig. 3), we further compared levels of immune markers in skin and blood of our patients. We quantified a panel of inflammatory molecules in respective lesional and non-lesional biopsies of our AD cohort using RT-PCR, and correlated them with serum OLINK levels (Fig. 4, Supplementary Figure S2). We found significant positive correlations for the general inflammation marker MMP12, the Th1/Th17 cytokine IL-12/IL-23p40, as well as Th1 (CXCL9), Th2 (CCL22) and Th17 (PI3/Elafin) markers (Fig. 4). Notably, these correlations were consistently found not only with lesional, but also with non-lesional skin (Fig. 4). IL-17A in skin was only upregulated in a subset of patients (Supplementary Figure S2i–j). When assessing skin with detectable IL-17, significant correlations of lesional (r = 0.53, p = 0.004), but not non-lesional skin were obtained with blood (r = 0.28, p = 0.29, data not shown).

### Multiple inflammatory pathways show a stronger enrichment in AD than in psoriasis

**Figure 5:**
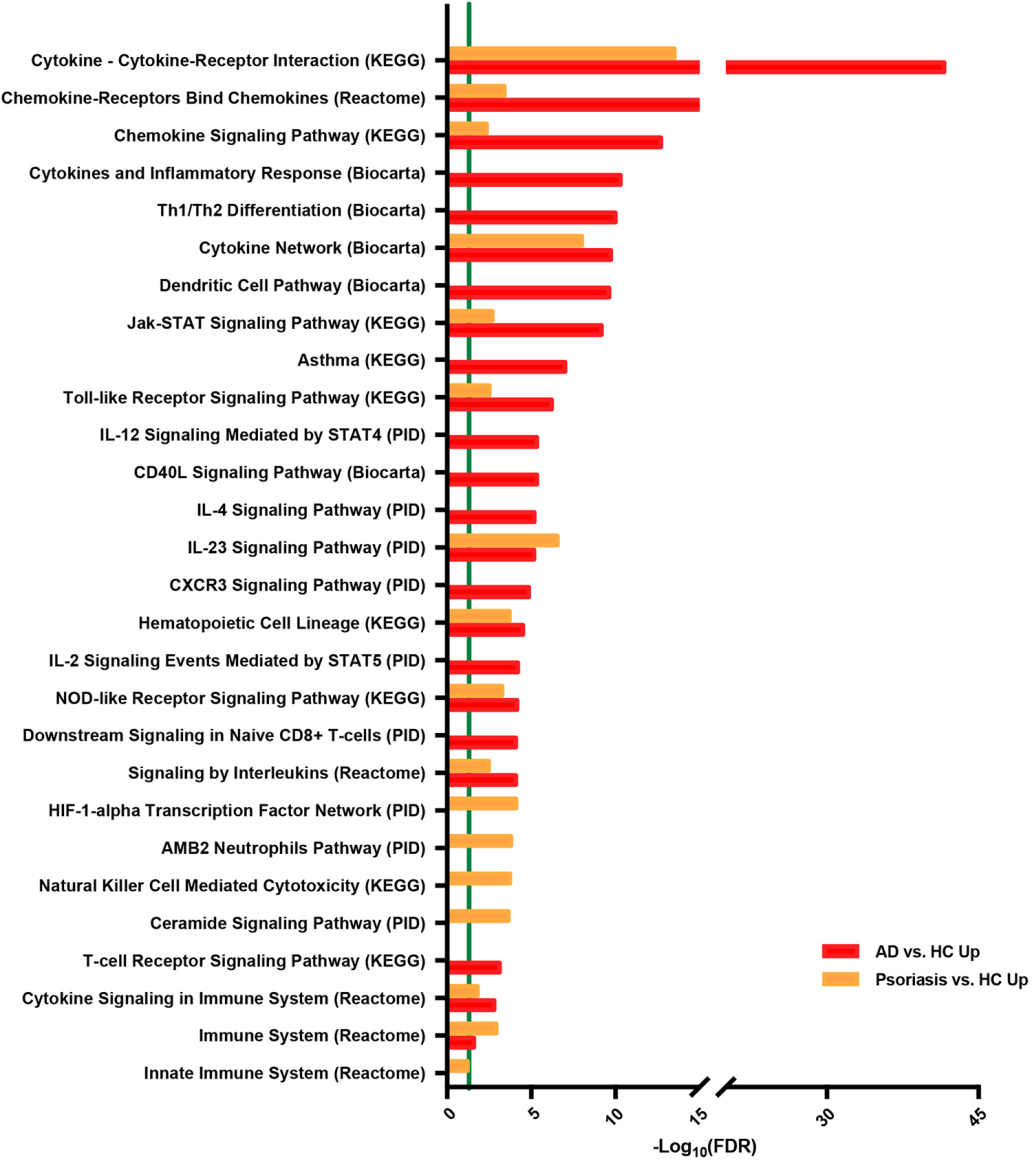
Pathway analysis. Selected pathways enriched in AD and psoriasis serum, compared to respective healthy controls; the green line indicates false discovery rate/FDR < 0.05.

We also conducted an enrichment analysis by using various published pathways (Kyoto Encyclopedia of Genes and Genomes/KEGG, Reactome Pathway Database, BioCarta, Pathway Interaction Database/PID) to compare AD and psoriasis blood proteomic profiles (Supplementary Table S5)^48–52^. Selected pathways significantly enriched in the serum of AD and/or psoriasis (in comparison to controls) are depicted in Fig. 5, with the green vertical line representing an FDR cutoff of 0.05. The top significantly enriched pathways in AD and psoriasis included Cytokine–Cytokine-Receptor Interaction, Chemokine–Chemokine-Receptor Binding, Chemokine Signaling, Cytokine Network, Jak-STAT Signaling, Toll-like Receptor Signaling, and IL-23 Signaling. Of these, only IL-23 signaling was more enriched in psoriasis, while all other pathways showed stronger enrichment in AD. Pathways exclusively enriched in AD included Cytokines and Inflammatory Responses, Th1/Th2 Differentiation, Dendritic Cell Pathway, Asthma, IL-12 STAT4 Signaling, CD40L Signaling, IL-4 Signaling, and CXCR3 Signaling, IL-2 STAT5 Signaling, CD8+ T-cell Signaling, and TCR Signaling. Pathways exclusively enriched in psoriasis included HIF1-alpha Transcription Factor Network, AMB2 Neutrophils Pathway, NK-Cell Mediated Cytotoxicity, Ceramide Signaling, and Innate Immune System (Reactome).

## Discussion

While several studies have described a limited set of biomarkers being increased in the blood of AD patients^31–33^, the current study is the first to investigate a broad array of immune and cardiovascular risk proteins in serum of moderate-to-severe AD patients, compared to psoriasis and controls. A large bulk of evidence in psoriasis suggests an increase in cardiovascular risk factors and associated cardiovascular comorbidity^53–55^, with similar data only recently emerging for AD^5,39^. The concept of increased cardiovascular risk and disease is supported by several epidemiological cohort studies^10,18,19,56^, case-control studies^13,15,16^ and population based surveys^12^. Using coronary computed tomography angiography, AD patients showed increases in coronary artery disease compared to healthy controls^16^. However, some other AD studies showed only marginal or no increased cardiovascular manifestations^11,14,57^, adding to the controversy as to whether AD is an independent risk factor for cardiovascular disease, mandating further investigation.

Recent studies in blood of severe AD patients showed significantly increased T-cell activation^25,30^ and increases in serum cytokines^31,33^ that correlated with clinical severity. Our study is the first to define inflammatory and cardiovascular risk proteins that are commonly upregulated in AD and psoriasis. These markers are largely associated with Th1 (IFN-γ, CXCL9, TNF-β, lymphotoxin-α), but also Th17 (CCL20) responses. Consistently, these immune axes are also upregulated in chronic skin lesions of both diseases^58^. IL-20 is involved in epidermal hyperplasia and inhibits keratinocyte differentiation^59^, but is also expressed in atherosclerotic plaques, and was shown to promote atherosclerosis in a mouse model^60^. IL-16, a chemo-attractant for CD4^+^ T-helper cells and myeloid cells, is also expressed in atherosclerotic plaques, but may have a plaque-stabilizing effect^61,62^. These factors, together with an environment of chronic/Th1-triggered inflammation^4,63^, with various degrees of Th17 activation^26,64,65^, suggest a potentially pro-atherogenic milieu in the blood of both psoriasis and AD patients that may directly impact endothelial cells^66^.

While we observed increases of several specific inflammatory and cardiovascular risk proteins only in psoriasis, including markers of coagulation, angiogenesis, endothelial activation and lipid metabolism, consistent with its well-established systemic inflammatory nature^67–69^, we also observed a large set of markers exclusively upregulated in AD. Consistent with skin data^4,5,58,70^, we found increases of Th1 (CXCL10, CXCL11), Th2 (IL-13, CCL13, CCL17, CCL11, IL-10), Th17/Th22 (S100A12) and Th1/Th17/Th22 (IL-12/IL-23p40) associated products in serum of AD patients. The fact that blood levels of the inflammatory marker MMP12 and several mediators from all T-helper-cell axes (CXCL9-Th1, CCL22-Th2, PI3-Th17, IL-12p40-Th1/Th17/Th22) correlated not only with lesional, but also with non-lesional skin, point to the critical role of systemic inflammation in immune abnormalities that AD already harbors in non-lesional skin. This systemic inflammation consists of a strong adaptive component, evidenced by increases in multiple factors involved in T-cell development and activation, such as CD40L, IL-7, CCL25, IL-2RB, IL-15RA, and CD6. IL-2RB is of special interest as it is part of the high-affinity IL-2 receptor, which is involved in transduction of mitogenic signals from IL-2.

IL-17C, produced by keratinocytes, endothelial cells, and leukocytes in the skin, can induce anti-microbial peptides in synergy with IL-22, TNF-α, and IL-1β^71^. In atherosclerotic plaques, smooth muscle cell-derived IL-17C plays a pro-atherogenic role by supporting recruitment of Th17 cells^72^. IL-17C and TNF-β, elevated in AD, have overlapping signaling pathways with IL-17A/F and TNF-α, which are thought to contribute to atherosclerosis in psoriasis^21,23^. Thus, synergistic effects on endothelial cells^73,74^ and other cell types need to be considered. TNFSF14/LIGHT, a pro-inflammatory cytokine associated with atherosclerosis^40^, plays crucial roles in T-cell homing into inflamed tissues^75^, and in the induction of matrix metalloproteinases/MMPs in macrophages^76,77^. Several MMPs, which are involved in tissue remodeling including atherosclerosis^78^, were also increased in AD serum (MMP-1, MMP-12, MMP-10).

When integrating many of these markers by using established lists of inflammatory pathways, we found enrichment of multiple pathways in AD to a much higher degree than in psoriasis. Enriched pathways included those also triggered by other atopic conditions, and particularly asthma (i.e. IL-4 immune signaling), also evidenced by the efficacy of specific Th2 targeting-strategies in both AD and asthma^79,80^. Many more immune pathways showed stronger enrichment in AD compared to psoriasis (e.g. cytokine-cytokine receptor interaction, chemokine signaling pathway, cytokines and inflammatory response, dendritic cell pathway, Th1/Th2 differentiation). These findings together with our past flow cytometry studies^30^, support a stronger systemic inflammation in AD compared to psoriasis.

Atherosclerosis is mediated by local inflammatory mediators including chemokines and their receptors, that are involved in the recruitment of inflammatory cells to the intima as an essential step in plaque development^81^. Such mediators were numerously increased in our AD cohort, including CCL4, CCL17, CCL28, CXCL5, CXCL10, and CX3CL1/fractalkine. CX3CL1/fractalkine is produced by endothelial cells, and is a strong chemoattractant for monocytes and lymphocytes, mediating their extravasation^82^. CCL4 and its receptor CCR5 have recently been demonstrated to play diverse roles in the inflammatory events underlying cardiovascular diseases and diabetes mellitus^83^. CXCL5 is increased in atherosclerosis, mediating a protective role in a mouse model by modulating macrophage activation^84^. CCL28 is chemotactic to T-cells, B-cells, and eosinophils to mucosal effector sites, and is increased in asthma^85^. CCL17 has been shown to drive atherosclerosis by restraining regulatory T-cell homeostasis^86^, and CXCL10 is associated with the severity of coronary artery disease^87^.

Several growth factors associated with atherosclerosis were also increased, such as the vascular growth factor VEGF-A^88^. Hepatocyte growth factor/HGF, produced by mesenchymal cells, is a biomarker of macroangiopathy^89^, and circulating HGF levels have been positively associated with stroke^90^. CD137, a co-stimulatory molecule expressed on activated T-cells, B-cells, DCs, as well as endothelial cells^91^, increases atherosclerosis in an ApoE(-/-) mouse model via leukocyte recruitment and inflammation^92^.

Taken together, we found multiple factors to be uniquely increased in AD that might be contributors of a pro-atherogenic burden in this disease. In line with recent publications^10,15,18,19^ showing increases in BMI in North American and Asian children and adults with AD, our cohort was also overweight. However, inflammatory mediators involved in atherosclerosis development (E-selectin, CCL7, IL16, PI3/elafin) were significantly correlated with AD severity/SCORAD, but not with BMI, strongly suggesting the contribution of cutaneous disease to cardiovascular morbidity.

Also, while associations with cardiovascular outcomes were reported in US and Asian studies^10,15,93^, only small increases in angina pectoris, arterial hypertension and peripheral arterial disease risks were found in a German cohort^14^. This European cohort also did not show increases in genetic risk factors for cardiovascular disease in AD^14^. One might speculate that varying degrees of cardiovascular disease across cohorts results from varying decades of chronic disease rather than due to shared genetic risks, but this assumption needs verification in the cohorts with robust increases in cardiovascular risk.

Our study poses several limitations. The subjects investigated (both healthy and AD populations) were overweight, potentially contributing to increases in inflammatory markers, and profiles might be different in a lean population. Another limitation is that while AD and healthy subjects were matched for BMI and age, psoriasis patients had higher BMIs, although our data were corrected for BMI (and also for other cardiovascular risk factors such as asthma, arterial hypertension, hypercholesterolemia, and diabetes mellitus). Larger future studies should be performed that will also include lean patients and control populations.

In sum, we have characterized a blood AD signature that is profoundly different from psoriasis. This profile helps to better understand cardiovascular risk in AD, and might also aid in identifying biomarkers to monitor therapeutic responses. Targeted therapeutic blockade of specific immune axes, e.g. Th17/IL-23 in psoriasis or Th2 in AD, is needed to assess the contribution of polar cytokine activation to overall systemic inflammation, and its effect on cardiovascular comorbidity and biomarkers. These studies should also assess whether biomarkers are modifiable risk factors responsive to treatment, as suggested by their decline with cyclosporine A treatment in severe chronic AD^33^.

## Methods

### Patients and samples

A cohort of 59 patients with moderate-to-severe AD (31 male and 28 female patients; mean age 40.5 years, range 18–72 years; mean SCORAD 54.1, range 34.5–89; mean blood eosinophils 6.91%, SD 4.94; median total serum IgE 2,412kU/L, IQR 5,028) was included in this study (Table 1, Supplementary Table S6). 20 patients (33.9%) reported to have mild asthma, of which 11 (18.6%) were on asthma treatment (inhaler); 9 patients (16.1%) reported seasonal allergies. 11 AD patients suffered from one or more of the following cardiovascular risk factors: Arterial hypertension (n = 8), diabetes mellitus (n = 4), or hypercholesterolemia (n = 7). Serum samples (n = 59) with corresponding lesional (n = 58) and nonlesional (n = 53) skin punch biopsies were collected. All methods were carried out in accordance with relevant guidelines and regulations. All experimental protocols were approved by the local institutional review boards (IRB of The Rockefeller University and the Icahn School of Medicine at Mount Sinai, both New York, NY). All patients gave written informed consent prior to inclusion. Lesional biopsies were obtained from chronic lesions, and non-lesional biopsies were taken from uninvolved skin in proximity but at least 4 cm away from lesional samples. 22 serum samples from moderate-to-severe psoriasis patients (18 male and 4 female patients; mean age 46.8 years, range 20–67 years; mean PASI 27.6, range 11.5–45.9) and 18 from healthy control subjects (12 male and 6 female subjects; mean age 41.3 years, 24–55 years) were obtained as comparators (Table 1, Supplementary Table S6). Washout periods prior to study inclusion were 4 weeks for systemic therapy (cyclosporine, oral steroids, azathioprine, mycophenolate mofetil and all other systemic immunosuppressants) and 2 weeks for phototherapy and topical corticosteroids for both psoriasis and atopic dermatitis. None of the subjects had any defined history of cardiovascular disease.

### OLINK multiplex assay

Serum samples were collected, centrifuged, and stored at −80 °C until further processing. Aliquots were analyzed with an OLINK Proseek® multiplex assay^40,94^, a proximity extension assay (PEA) technology with oligonucleotide-labeled antibody probe pairs that bind to their respective targets^41^. Upon binding of antibody pairs to their respective targets, DNA reporter molecules bound to the antibodies gave rise to new DNA amplicons with each ID-barcoding their respective antigens. The amplicons were subsequently quantified using a Fluidigm BioMark^TM^ HD real-time PCR platform^40^. Serum was analyzed using Inflammation I, cardiovascular disease/CVD II, and CVD III multiplex panels, which contain a broad array of established and exploratory markers^40^. OLINK data per subject are given in Supplementary Table S6.

### Skin RT-PCR

Real time-PCR was performed on AD-related genes as described previously^95,96^. Results were normalized to the housekeeping gene *hARP* and log2-transformed for analysis. Primers and probes used are listed in Supplementary Table S7.

### Statistical analysis

Statistical analysis was carried out using R-language (R-project.org) and packages available through the Bioconductor Project (www.bioconductor.org).

#### Quantification of Protein levels

Quality control of OLINK chip data was carried out using their standard quality control pipeline (QC)^40,94^. A minor number of samples in each panel were excluded after this quality control procedure. A small batch effect (explaining 8.4% of the variance) was observed corresponding to the processing times. This plate effect was estimated through a linear model using a set of intra-plate control samples that were repeated in each plate. We corroborate that the batch was successfully adjusted for the set of 7 intra-plate samples (showed in duplicates in Supplementary Table S6) presented in the statistical analysis for this paper.

#### Analysis of Protein profiles

Protein expression profiles were modeled using linear models for high-throughput data on R’s *limma* framework. The model included Disease as a factor and covariates Age, Gender and BMI. Comparison between groups were estimated using an empirical Bayesian method^97^ available in the *limma* package; this uses the variance across all genes to estimate per gene variance. After model estimation using residual maximum likelihood algorithms, hypothesis testing was conducted for comparisons of interest using contrasts under the general framework for linear models in the *limma* package. P-values from the moderated t-test were adjusted for multiple hypotheses using the Benjamini–Hochberg procedure, which controls the FDR (false discovery rate). A sensitivity analysis including asthma and cardiovascular (CVD) risk outcomes as a covariate was also carried out. Covariates with missing values were imputed as the mean (age, BMI) and as “not present” for binary comorbidities. Sensitivity analysis showed no departure from the attained conclusions due to imputation. Comparison of protein profiles among groups was carried out using linear models for high-throughput data on R’s *limma* framework. Protein annotations, as well as Gene-Protein relationships were obtained by using UniPro IDs and R’s *AnnotationDbi* package.

#### RT-PCR data

Ct values were derived by normalizing Ct values to an endogenous control gene (hARP). Values under the limit of detection (LOD) were substituted by the 20% of the minimum value above the LOD. Data was log2-tranformed prior to analysis.

Correlation between skin mRNA and protein profiles was evaluated using Pearson and Spearman correlation coefficients on log2-transformed levels. Data is presented in scatterplots with estimated linear regression and 95% confidence interval.

#### Pathway Enrichment Analysis

Gene set over-representation analysis was performed using XGR software^98^ based on functional category including KEGG^99^, BioCarta^100^, REACTOME^101^, PID^51^ and MSigDB^102^. The significance of the overlaps was assessed using FDR < 0.05.

### Data availability

All data generated or analyzed during this study are included in this published article (and its Supplementary Information Files).

## Electronic supplementary material

Supplementary Material

Supplementary Table S1

Supplementary Table S2

Supplementary Table S3

Supplementary Table S4

Supplementary Table S5

Supplementary Table S6
