# Author Correction: The atopic dermatitis blood signature is characterized by increases in inflammatory and cardiovascular risk proteins

**DOI:** 10.1038/s41598-018-26378-5

**Published:** 2018-05-29

**Authors:** Patrick M. Brunner, Mayte Suárez-Fariñas, Helen He, Kunal Malik, Huei-Chi Wen, Juana Gonzalez, Tom Chih-Chieh Chan, Yeriel Estrada, Xiuzhong Zheng, Saakshi Khattri, Annunziata Dattola, James G. Krueger, Emma Guttman-Yassky

**Affiliations:** 1 The Laboratory for Investigative Dermatology, The Rockefeller University, New York, NY USA; 2 Department of Dermatology and the Laboratory for Inflammatory Skin Diseases, Icahn School of Medicine at Mount Sinai, New York, NY USA; 3 Department of Population Health Science and Policy, Icahn School of Medicine at Mount Sinai, New York, NY USA; 4 Department of Genetics and Genomics Science, Icahn Institute for Genomics and Multiscale Biology, New York, NY USA

Correction to: *Scientific Reports* 10.1038/s41598-017-09207-z, published online 18 August 2017

The original version of this Article contained a typographical error in the spelling of the author Annunziata Dattola, which was incorrectly given as Nancy Dattola. This has now been corrected in the PDF and HTML versions of the Article, and in the accompanying supplementary material.

The Author Contributions section now reads:

P.M.B., M.S.F., J.G., A.D., J.G.K. and E.G.Y. designed the research study. K.M., H.C.W., T.C.C., Y.E., X.Z. and S.K. acquired the data, and M.S.F., H.H. and H.C.W. performed statistical analyses. P.M.B., J.G.K. and E.G.Y. wrote the manuscript. All authors critically revised the manuscript and approved its final version.

