# Supplementary material for: The atopic dermatitis blood signature is characterized by increases in inflammatory and cardiovascular risk proteins
Source: Sci Rep. 2017 Aug 18;7:8707. doi: 10.1038/s41598-017-09207-z (PMC5562859; doi:10.1038/s41598-017-09207-z)
Supplement: Supplementary file 1 — Supplementary Material [file 41598_2017_9207_MOESM1_ESM.docx]

**SUPPLEMENTARY MATERIAL**

**The atopic dermatitis blood signature is characterized by increases in inflammatory and cardiovascular risk proteins**

Patrick M. Brunner MD^1^, Mayte Suárez-Fariñas PhD^2,3,4^, Helen He BS^2^, Kunal Malik BA^2^, Huei-Chi Wen MD, PhD^2^, Juana Gonzalez PhD^1^, Tom Chih-Chieh Chan MD^2^, Yeriel Estrada BS^2^, Xiuzhong Zheng MSc^1^, Saakshi Khattri MD^2^, Annunziata Dattola, MD^1^, James G. Krueger MD, PhD^1^, Emma Guttman-Yassky MD, PhD^1,2^

^1^ The Laboratory for Investigative Dermatology, The Rockefeller University, New York, NY, USA

^2^ Department of Dermatology and the Laboratory for Inflammatory Skin Diseases, Icahn School of Medicine at Mount Sinai, New York, NY, USA

^3^ Department of Population Health Science and Policy, Icahn School of Medicine at Mount Sinai, New York, NY, USA

^4^ Department of Genetics and Genomics Science, Icahn Institute for Genomics and Multiscale Biology, New York, NY, USA

**
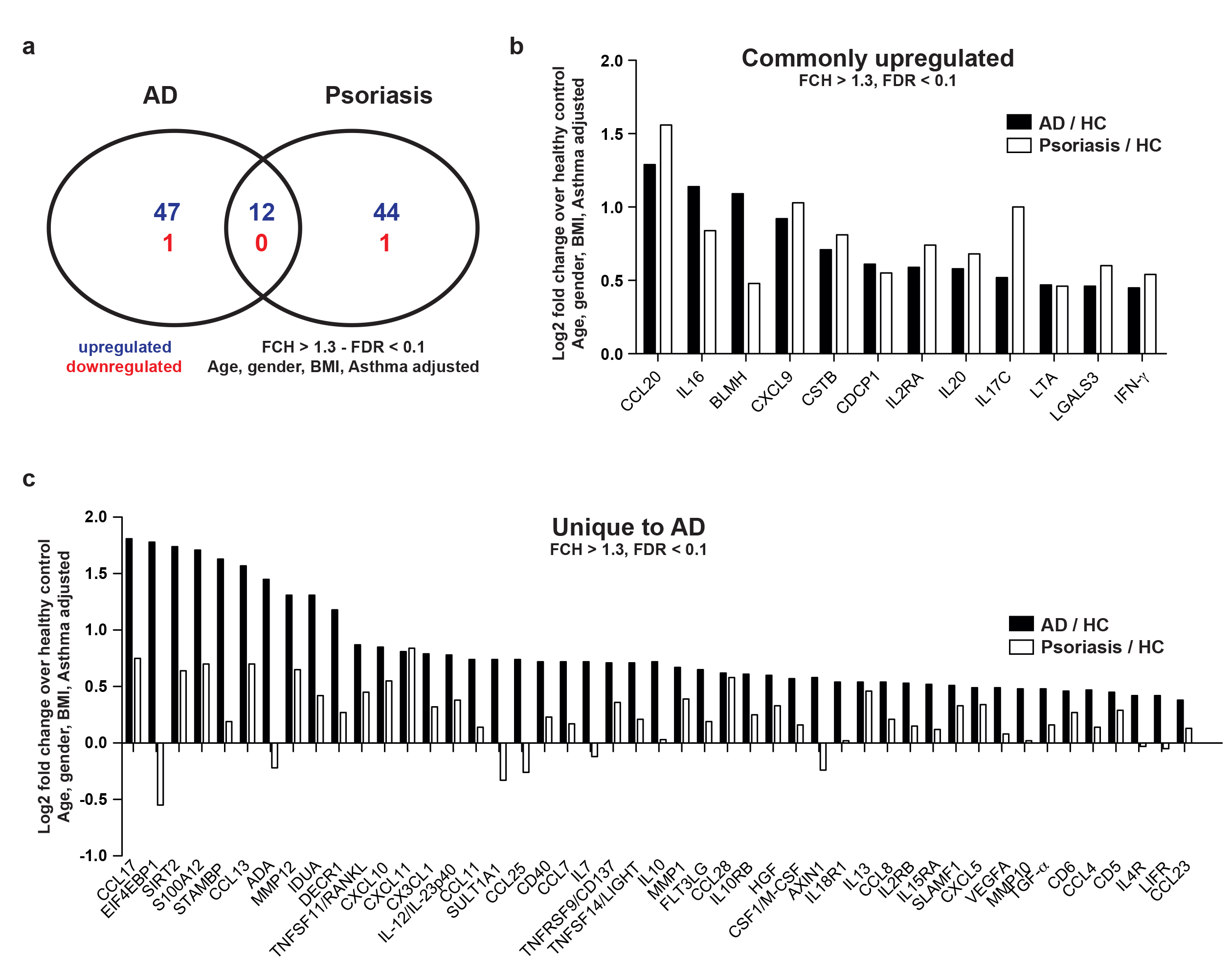
**

**Figure S1.** Venn diagrams of regulated serum proteins in AD and psoriasis compared to healthy controls, adjusted for age/gender/BMI/asthma and cardiovascular (CV) risk factors (i.e. arterial hypertension, hypercholesterolemia, diabetes mellitus) **(a).** Markers that were significantly upregulated (FCH>1.3, FDR<0.1) in both AD and psoriasis **(b)**, or only in AD **(c)** are depicted as log2 fold change over healthy control serum with their 95% confidence interval.

**
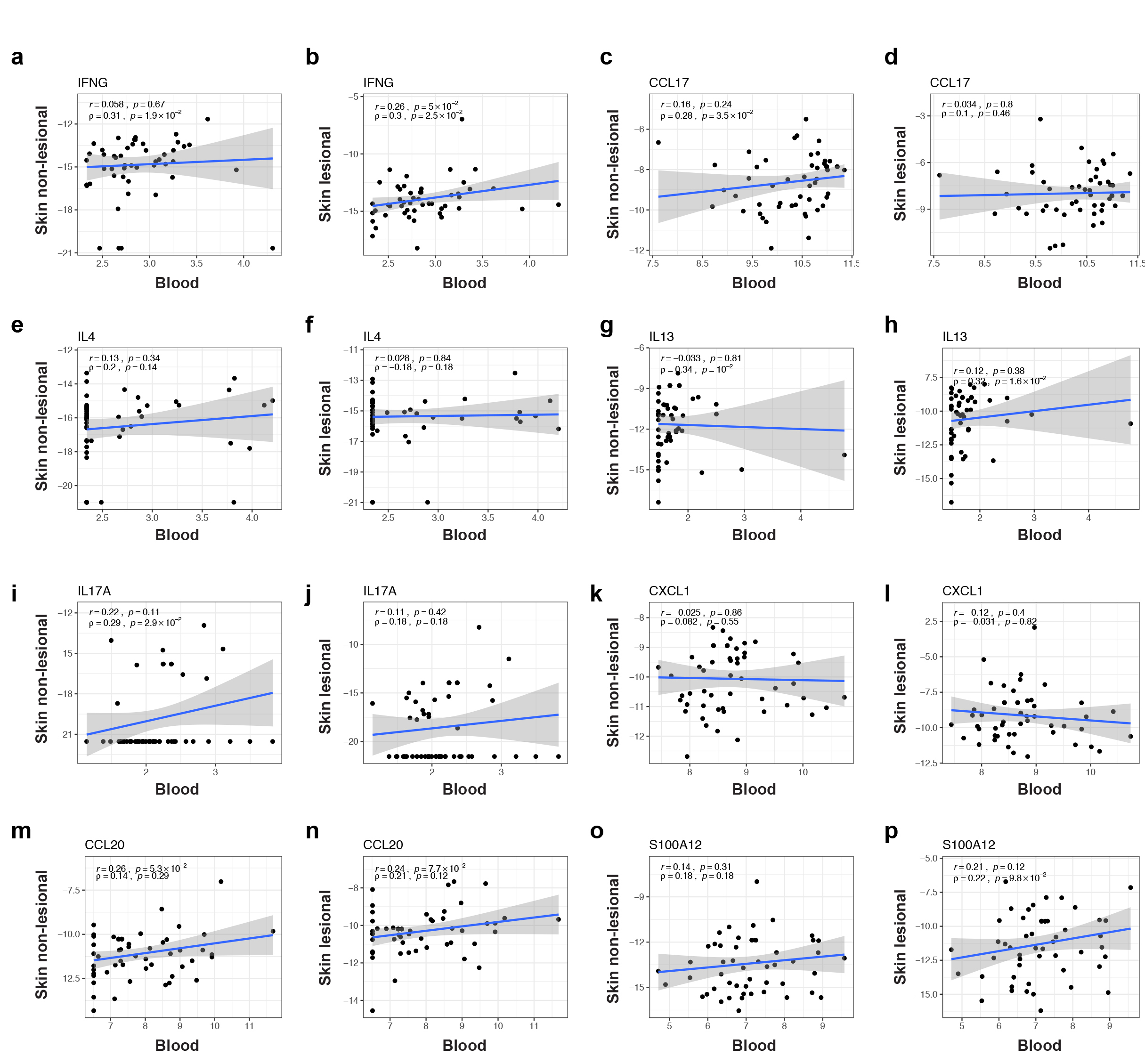
**

**Figure S2.** Correlation plots of selected serum protein levels with their corresponding lesional and non-lesional skin mRNA levels; scatterplots with estimated linear regression and 95% confidence interval; *r Pearson correlation; ρ Spearman correlation.*

**Supplementary Table S1.** Regulation of serum proteins, adjusted for age and gender (see Excel file).

**Supplementary Table S2.** Regulation of serum proteins, adjusted for age, gender and body mass index/BMI (see Excel file).

**Supplementary Table S3.** Regulation of serum proteins, adjusted for age, gender, as well as asthma and cardiovascular risk factors (arterial hypertension, hypercholesterolemia, diabetes mellitus) (see Excel file).

**Supplementary Table S4.** Correlation of serum markers with SCORAD and body surface area (BSA); Pearson Correlation Coefficient and p values (see Excel file).

**Supplementary Table S5.** Pathway enrichment analysis (see Excel file).

**Supplementary Table S6.** Demographics, baseline characteristics, comorbid conditions and OLINK data per patient (see Excel file).

| **Gene symbol** | **Sequence** |
| --- | --- |
| **RPLP0/hARP** | Forward CGCTGCTGAACATGCTCAA |
|  | Reverse TGTCGAACACCTGCTGGATG |
|  | Probe 6-FAM-TCCCCCTTCTCCTTTGGGCTGG-2TAMRA |
|  | **Assay ID** |
| **IL17A** | Hs00174383_m1 |
| **IL13** | Hs00174379_m1 |
| **CCL17** | Hs00171074_m1 |
| **IFN gamma** | Hs00989291_m1 |
| **IL12/IL23p40 (IL12B)** | Hs01011518_m1 |
| **PI3/Elafin** | Hs00160066_m1 |
| **MMP12** | Hs00159178_m1 |
| **IL4** | Hs00174122_m1 |
| **CXCL1** | Hs00236937_m1 |
| **CCL20** | Hs00355476_m1 |
| **S100A12** | Hs00942835_g1 |
| **CCL22** | Hs01574247_m1 |
| **CXCL9** | Hs00171065_m1 |

**Supplementary Table S7.** Primers and probes used for qRT-PCR.
